# Perceived support needs of novice nurses working in emergency departments of selected public hospitals in Tshwane, South Africa

**DOI:** 10.1016/j.afjem.2025.100889

**Published:** 2025-07-23

**Authors:** Kapari Mashao, Celia Filmalter, Tanya Heyns

**Affiliations:** Department of Nursing Science, Faculty of Health Sciences, University of Pretoria, Pretoria, South Africa

**Keywords:** Emergency department, Novice nurse, Support needs, Orientation programme, Preceptor

## Abstract

**Introduction:**

Patients present with diverse healthcare needs in emergency departments, necessitating nurses to have diverse clinical competencies to function effectively. Novice nurses in emergency departments may be ill-equipped to handle responsibilities and workload, necessitating adequate support for a smooth transition. However, providing practical support requires a comprehensive understanding of support needs.

**Methods:**

This descriptive qualitative study investigated the perceived support needs of novice nurses working in emergency departments. Fifteen participants were purposively selected from three selected public hospitals in Tshwane, South Africa. Semi structured, face-to-face interviews were conducted between September and November 2023. The interviews were audio-recorded, and the transcripts were thematically analysed via ATLASti Version 24.

**Results:**

Three key themes emerged to support the transition of novice nurses in emergency departments: a dedicated orientation programme, a dedicated preceptor, and a supportive culture to facilitate the transition to emergency departments. A dedicated orientation programme encompasses essential components, including emergency equipment, triage, basic life support skills, admission and treatment protocols, and specific competencies. The participants emphasised the value of dedicated preceptors, trained and experienced professionals who offer guidance, feedback and remedial support. Additionally, the importance of a supportive culture, particularly one that fosters a positive learning environment and provides emotional support to ease the transition process, was highlighted.

**Conclusion:**

The successful transition of novice nurses in emergency departments is a cost-effective way of improving job satisfaction and retention. However, novice nurses working in emergency departments have diverse support needs. Thus, ensuring a successful transition requires understanding these nurses’ support needs. This study revealed that establishing a structured orientation programme, assigning trained preceptors and fostering a supportive culture are critical in facilitating the successful transition of novice nurses to emergency departments. These strategies can address novice nurses’ support needs by improving competence and patient care outcomes and promoting confidence, emotional well-being, job satisfaction and retention. Realising the support needs of novice nurses may inform the development of evidence-based transition interventions that offer support in specific ED contexts.

## African Relevance

• Nurses in emergency departments are often the first to receive patients; hence, support is imperative.
• African countries have fewer emergency nurses due to limited training institutions.
• The emergency departments of African countries have more novice nurses who need adequate competencies to function effectively in these environments.
• This study investigated the support needs of novice nurses in South African emergency departments, which may be relevant to other African countries.
• Understanding the needs of novice nurses transitioning to emergency departments may provide hospital authorities with evidence to structure orientation and support programs.

## Introduction

Emergency departments (EDs) are specialised, unique, fast-paced, multifaceted, and unpredictable environments [1]. The complexity and diversity of patient care needs in EDs demand that nurses with adequate competencies make high-level clinical decisions [2]. Failure of nurses to recognise and respond to emergencies may increase the incidence of morbidity and mortality [3,4]. However, a global nurse shortage, particularly in EDs, leads to these environments being staffed by a mix of untrained and trained emergency nurses [5].

Trained emergency nurses in some settings formally acquire adequate ED competencies through postgraduate emergency nursing programmes [6]. Nurses who attend such programmes are prepared to take care of foreseeable emergencies, are skilled at identifying life-threatening problems, prioritise the urgency of care and rapidly and effectively carry out resuscitative measures and other treatments [7,8]. However, few African institutions offer postgraduate emergency nursing programmes, resulting in a limited number of trained emergency nurses [9]. To curb ED nurse shortages, authorities employ untrained emergency nurses to work in EDs [10].

In some high-income settings, including Canada [11,12], Australia [13] and New Zealand [14], nurses working in EDs are required to have advanced knowledge and skills; hence, one or more years of working experience as nurses are required before working in EDs [2]. These countries’ health system capacity, nursing education standards, and workforce supply structures make these ED employment requirements possible [[12], [13], [14]]. The limited number of trained emergency nurses, coupled with resignations and retirements in many EDs, particularly in low and middle income countries such as South Africa, has created a situation where more novice nurses work in EDs [[16], [17], [18]].

Novice nurses are defined as nurses who are encountering a completely different working environment that demands competencies distinct from those required in previous roles [19]. These nurses have little or no experience in the new environment; hence, new competencies need to be developed to adapt and perform effectively in the new environment [19,20]. Therefore, regardless of whether nurses are newly graduated or experienced, they are considered novice nurses once they start working in EDs [17]. In the ED, novice nurses are exposed to various patient populations and emergency conditions [21]. The competencies required by nurses working in EDs are similar to those acquired by trained emergency nurses, which include identifying and managing life-threatening conditions and prioritising and providing effective interventions, including resuscitations [8,22,23]. However, the transitional support of novice nurses working in EDs varies from a lack of support to short orientation programmes focused mainly on hospitals’ nursing regulations and procedures and basic nursing operations [[24], [25], [26]]. Hence, most novice nurses have insufficiently developed competencies to function optimally in EDs, which makes them unprepared to deal with emergencies [23].

Additionally, novice nurses in the ED are confronted with challenges, including heavy workloads, unique responsibilities, limited resources, and the demand for interpersonal relationships [1,27]. Novice nurses are likely to be the first to recognise and respond to emergencies [23]. The limited experience of these nurses may lead to a lack of confidence, negative emotions, and poor patient care, including increased medical errors and poor retention [17,23]; therefore, transitioning to EDs can be challenging and stressful for novice nurses [20].

In several studies, novice nurses reported being unprepared for ED duties and workloads yet were expected to perform similar responsibilities and demonstrate the same competence as experienced ED nurses [1,24,28]. However, the limited basic emergency knowledge and skills and inadequate ED exposure that nurses acquire in undergraduate nursing programmes are insufficient for effective ED functioning [28,29]. The evidence suggests that adequate transitional support can positively impact novice nurses’ clinical learning and socialisation, job satisfaction, patient outcomes and retention [26,30]. However, ensuring adequate support requires more than skill acquisition and knowledge advancement [31]. Globally, novice nurses’ transitional support in EDs includes continuing professional development, orientation programmes, nurse residency programmes and transition programmes to facilitate the transition of novice nurses to EDs [24,[32], [33], [34]].

Inadequate support for novice nurses can hinder clinical progression and potential assimilation into EDs, negatively impacting transition outcomes [35]. Without adequate support, novice nurses may experience emotional and mental pressures, leading to frustration, decreased confidence, feelings of incompetency, poor patient outcomes, job dissatisfaction and occupational stress [28]. The demanding nature of ED environments often harms novice nurses’ health, as they present with confusion, stress, anxiety, decreased self-esteem, and feelings of inadequacy [30]. When appropriate support is lacking, novice nurses may underperform, leading to decreased job satisfaction, lower retention, and poor patient outcomes [17,35]. Over time, these effects may lead novice nurses to leave the profession because they cannot adapt to their role [36].

Adequate transitional support for novice nurses in EDs has been proven to lower stress levels and improve clinical competence, critical thinking, self-confidence, retention, job satisfaction and patient outcomes [25,33]. To enhance a smooth transition process, an understanding of novice nurses’ needs is needed [4,17]. This study aimed to explore and elucidate the perceived support needs of novice nurses employed in EDs.

## Methods

### Study design

This descriptive, qualitative study investigated how novice nurses perceive their support needs in EDs. The research is reported following the CONSolidated Criteria for Reporting Qualitative Research (COREQ) [37].

### Setting

The study was conducted in three EDs in public hospitals in Tshwane Central Health District, South Africa. The three hospitals (A, B & C) were purposively selected because they are accredited training hospitals supporting undergraduate and postgraduate students enrolled at the university. Each ED comprises different categories of nursing staff, including novice nurses (Table 1). Between three and four novice nurses are employed annually by the three hospitals on average.

**Table 1:** Summary of hospital Emergency Department settings in the three included hospitals.

Hospital	Category hospital	Emergency department		
		Total nurses (Count)	Nurses per shift (Count)	Patients/24 h (Count)
A	Central (832 beds)	40	17 to 20	70 to 80
B	Provincial tertiary (857 beds)	36	10 to 12	80 to 90
C	District hospital (200 beds)	24	6 to 8	45 to 50

The three hospitals vary in the level of care provided, with some offering mental healthcare services in their EDs, and Hospitals A and B providing more specialist-level services [38].

In South Africa, there are no ED-specific courses during undergraduate programmes, nor nurse residency programmes that prepare nurses for the ED environment. Novice nurses in the selected hospitals are offered induction programs (between one and two weeks facilitated by hospital staff development clinical facilitators), and short orientation programmes (from one to five days offered by emergency department managers or nurse shift leaders). Although each hospital ED has a clinical facilitator, these facilitators are assigned to different hospital wards, focusing on in-service training rather than continuous support to novice nurses in EDs.

### Participant selection

Novice nurses were purposively sampled if they had worked in an ED for at least nine months and no more than three years [39]. Purposive sampling was used to ensure the selection of participants with ED transition experience and could provide good insights into the perceived support needs of novice nurses working in the ED. Five novice nurses from each hospital were recruited to maximise representation. In total, 15 participants were recruited for the study. The hospitals’ chief executive officers and ED operational managers were contacted via email. The lead author (KM) then met face-to-face with the novice nurses and presented the study. Voluntary participation was emphasised, and all participants were informed that they could withdraw without penalty.

### Data collection

KM conducted face-to-face interviews between September and November 2023. An interview guide was used to ensure consistency when interviewing participants. A pilot interview was conducted to evaluate the structure and flow of the interview and the effectiveness of the questions, as well as to refine the interviewer's interview skills and confidence [40]. No changes were made; therefore, the data were analysed.

The author overcame the researcher-participant power play by using icebreakers and pleasantries before the interviews, including sharing ED interests. Moreover, the author ensured that they wore the nursing uniform when conducting the interviews, owing to the interview venue. Field notes were taken, and the interviews were audio recorded. The interviews were conducted at times and venues convenient for the participants and lasted between 30 and 60 min. All 15 interviews were conducted face-to-face in the private and comfortable rooms of the selected EDs. The interview time varied: some participants were interviewed after work shifts, others utilised lunch times, and others were interviewed before starting work shifts (particularly those working night shifts). One question was posed: “On the basis of your experience, what are your perceptions regarding the support needs of novice nurses working in the ED?” Probing questions were subsequently asked to gather detailed information and clarify responses [41]. During the interviews, pseudonyms were used to increase confidentiality. Three additional interviews were conducted to ascertain data saturation, and no new information was identified. Member checking was performed after all the interviews were transcribed. Data saturation was obtained after 12 interviews. The recordings were transcribed and returned to the participants for comments, and no changes were made. The participants acknowledged and confirmed the accuracy of the data.

### Ethical approval

Ethical approval was granted by the Research Ethics Committee of the University of Pretoria (245/2023), and further permission for doing the research was obtained from the National Health Research Database (GP_202,308_083). Written and verbal consent was obtained from each participant before the interviews.

### Data analysis

The authors worked with an independent coder. Thematic data analysis described by Braun and Clarke [42] was used. In step one, familiarisation was achieved by reading and rereading the transcripts. In step two, the data were inductively analysed via ATLASti Version 24 qualitative software following a seven-step process. After completing the data analysis, the independent coder and authors discussed and agreed on the final themes and categories.

### Trustworthiness

We applied different strategies to enhance trustworthiness. The credibility of the data was enhanced by space data triangulation, peer debriefing and member checking [41]. Space triangulation included collecting data from the three EDs of the selected hospitals. The authors and an independent coder used peer debriefing to enhance credibility. The credibility of the data was further enhanced by sending transcripts to participants for confirmation and approval of the data. Dependability is enhanced by use of the COREQ checklist to ensure transparency [37]. Transferability was enhanced by clearly describing the study setting, sampling, data collection and analysis methods to understand the research process. Authenticity was demonstrated by representing participants’ perceptions via quote excerpts.

## Results

All 15 novice nurses who accepted the invitation participated, and this included men (*n* = 5) and women (*n* = 10), with a mean working experience of 18.4 months (SD=10.24 months). Most of the participants had diplomas (*n* = 12) in nursing. Three themes and nine related categories were generated from the semi structured data (Table 2). Themes include dedicated orientation programmes, dedicated preceptors and a supportive culture to facilitate transition in the ED. The participants voiced that formal support to foster transition in the ED can support them, as evidenced in Table 2.

**Table 2:** Summary of themes and categories.

Themes	Theme 1 Dedicated orientation programmes	Theme 2 Dedicated preceptors	Theme 3 Supportive culture to facilitate transition in the ED
**Categories**	Emergency equipment Triage Basic life support skills Admission and treatment protocols Specific competencies	Availability of trained and experienced preceptor Feedback and remedial to facilitate learning	3.1 A positive learning environment Emotional support

*Theme 1: Dedicated orientation programmes*

The participants described EDs as ‘*an overwhelming environment’,* requiring a dedicated orientation programme to enhance the smooth transition of novice nurses to the ED:

*“…there needs to be a programme for new [novice] nurses who come in the emergency department. For some people, this can be very overwhelming because it is fast paced…” (P7)*

*Category 1.1: Emergency equipment*

The participants indicated that they were never exposed to some of the emergency equipment used in the ED, which ‘*frustrated’* them. The participants expressed the need for orientation on ED-specific equipment.

*“…in terms of machines [equipment] used during my [novice nurse] training, we never truly did ECGs [electrocardiograms]… Not all of us know how to operate the machine…” (P11)*

*“…what I struggle most with is that where I worked before, we didn’t have the blood gas machine…” (P2)*

*Category 1.2: Triage*

The participants highlighted the importance of orientation concerning triage.

*“…As new [novice] nurses, we need detailed training on triage… clear instructions and directives on who can triage a patient…” (P13)*

*Category 1.3: Basic life support skills (BLS)*

The participants agreed that cardiopulmonary resuscitation (CPR) is frequently performed in EDs and that they lack confidence. They expressed a need for training on the BLS.

*“… Resuscitation [CPR] is one of the most important things that we must understand and know because it happens almost every day here [ED]…” (P4)*

The participants strongly recommended that the BLS international accredited course be a standard training offered to novice nurses:

*“… I would also suggest that BLS be recommended as a standardised course…” (P6)*

*Category 1.4: Admission and treatment protocols*

The participants indicated that admission and treatment protocols should be prerequisites for any ED environment:

*“…There should be protocols providing guidelines for each condition that are commonly managed in the unit [ED] …taking pictures of a prepared ventilator helped me…taking a short video when somebody's doing a skill and going to Google and comparing…so sometimes the only thing that was helping is to go to Google…” (P4)*

*Category 1.5: Specific competencies*

The participants highlighted that “*patients of all ages*” are admitted and managed.

*“… Like how to assess and manage DKA [diabetes ketoacidosis] in the emergency department… also to work with mental healthcare users… and paediatrics. Some of the patients are pregnant…” (P14)*

The participants expressed the need for specific competencies in managing patients presenting with common conditions to the ED, as supported by quotes in the Supplemental material. In addition, the participants indicated that they needed training on ECG interpretation, management of a patient on mechanical ventilation, arterial blood gas analysis, intravenous and urinary catheter insertion, chest X-ray interpretation, suturing, assisting with intubation, medication, documentation, and interpersonal skills (Supplemental material).

*Theme 2: Dedicated preceptors*

The participants expressed a need for a dedicated preceptor to guide and support them through their transition.

*Category 2.1: Availability of trained and experienced preceptors*

The participants indicated that they wished to be paired with a preceptor with “knowledge and experience” (P3), supported by the following:

*“…I [novice nurse] would love to have someone like a preceptor… I think if I had a preceptor, it would have facilitated my transition a lot better and a lot quicker… someone that has worked in an emergency and has ED experience.” (P1)*

Category 2.2: Feedback and remedial support to facilitate learning

The participants highlighted the importance of the feedback and remedial feedback required from preceptors:

*“…to have someone who gives you feedback on your performance… it would have helped me improve…I would know my faults and the feedback that I get from my preceptor…” (P1)*

*Theme 3: Supportive culture to facilitate transition in the emergency department*

A supportive culture to facilitate learning included a positive learning environment and emotional support.

*Category 3.1: A positive learning environment*

The participants expressed their feelings of frustration when working in the ED environment and their acceptance by ED staff:

*“…if we [novice nurses] can get proper teamwork, support from our seniors [experienced nurses] and support each other in the unit, I think it will be much easier…it will make the work environment to become calmer and more conducive for our learning…” (P2)*

*“…I would recommend that the personnel [healthcare professionals] take more time and become impatient with the new personnel [novice nurses] …” (P8)*

*Category 3.2: Emotional support*

The participants reported having experienced a lack of emotional support:

*“…Working in the emergency department is not for the faint-hearted; therefore, it would be best for novice nurses to have support groups in order to discuss their emotional challenges in a safe environment…” (P15).*

*“…Also, to check the emotions and how we cope because it is trauma events here [ED]. Some we don’t cope, I had nightmares at night, they [ED managers] couldn’t pick up that…you get so much of a stress. Then when you [novice nurse] get home, you don’t get someone to talk to or someone who understand your work…” (P6).*

## Discussion

The focus of transition support to novice nurses working in the ED has been mainly on professional knowledge and skills. Our findings suggest that, in addition to educational needs, novice nurses require healthy and supportive learning and working environments for successful transitions [43,44]. Our findings suggest that novice nurses working in the EDs of public hospitals in South Africa require specialised training before they feel comfortable in their new environment. However, healthcare facilities only provide a general orientation to novice nurses to increase their competencies and confidence during their initial employment [45,46]. The general orientation includes classroom instruction, preceptor support and rotation through different hospital units. Nonetheless, this orientation does not prepare novice nurses for ED environments. Nurses in EDs care for patients of all age groups [22], with varying degrees of clinical urgency and severity. Nurses must prioritise time-critical interventions; therefore, learning these skills requires an extended orientation specific to the ED environment [1].

The participants in our study highlighted three supportive needs for novice nurses working in the ED: dedicated orientation programmes, dedicated preceptors, and a supportive culture to facilitate transition in the ED. Globally, many EDs offer orientation programmes to ease the transition of novice nurses [47]. Orientation programmes have proven effective in helping novice nurses better and more rapidly adapt to the clinical environment, improve retention and allow for smooth transition to practice [16,24,47]. Our study, which is consistent with previous studies, indicated that a dedicated orientation programme should include training in emergency equipment, triage, basic life support skills, admission and treatment protocols and specific competencies [32,47].

The range of health problems encountered in EDs requires nurses to be knowledgeable in operating ED-specific equipment [48]. Nurses working in EDs are expected to use different equipment for diagnosis and monitoring [48]. Consistent with these findings, our study revealed that novice nurses in EDs need to be knowledgeable and skilled in operating equipment such as electrocardiogram machines [45], mechanical ventilators [48], and arterial blood gas analysers [49]. Knowing how to effectively operate the most frequently used equipment, including electrocardiogram machines and defibrillators, can enable novice nurses to identify conditions, such as cardiac arrest, that require prompt interventions [34]. Hence, patient safety and timely access to care in the ED, particularly in triage, rely on this emergency equipment.

Triage is integral to the ED, yet nurses often face time pressure with limited knowledge and skills [50]. Hence, equipping novice nurses with the necessary triage competencies will promote patient safety in the ED. The roles and skills of triage nurses include conducting rapid clinical assessments and assessing urgency and severity in unpredictable situations [34]. The novice nurses in our study expressed a need to be competent in triage. Education on the triage process, protocols, and triage scale may improve triage accuracy, processing and decision making [34], as evidenced in South Africa [4,50] and Egypt [51].

Basic life support (BLS) is the foundation of saving life after cardiac arrest [52]. The fundamental aspects of BLS, among others, include recognising cardiac arrest and performing cardiopulmonary resuscitation (CPR) [52,53]. Cardiopulmonary resuscitation is often performed in EDs and requires specialised resuscitation skills [54]. BLS and advanced cardiac life support training should be continuously updated to align with current evidence-based practices [4,55]. Consistent with our findings, several studies on BLS skills have been conducted in countries such as Indonesia [56], China [24], Kenya [28], Egypt [57], and South Africa [4,50], reporting that novice nurses have inadequate knowledge and skill deficits in BLS. Education and refresher training for these nurses are thus vital. In a fast-paced environment such as the ED, protocols may help novice nurses acquire knowledge of ED processes and functions, including BLS skills.

Protocols guide healthcare professionals in making decisions and actions related to patient care [23]. The novice nurses in our study highlighted the need for protocols targeted at ED admission and treatment, as well as for patient referral protocols, which are supported by the South African Department of Health [38]. Similar findings were reported in Spain, where novice nurses reported a lack of easily accessible validated reference materials, which led to the use of informal resources such as mobile phones and tablets [58]. ED protocols may be helpful for novice nurses in busy and understaffed EDs, although specific competencies should be emphasised.

Evidence shows that nurse competency can influence patient outcomes [4]. Hence, the novice nurses in our study expressed a need for training in specific ED competencies (Supplemental material). Consistent with our findings, studies have shown that ED competencies, such as assessing and managing patients and procedures, may improve competency and enhance the transition of novices in the ED [4,28,59]. Aside from patient assessment and procedures, novice nurses also need to address the increasing presentation of mental healthcare users to EDs [60]. A study from Sweden revealed that ED nurses need knowledge and training to manage patients presenting with mental healthcare challenges [61]. A previous study reported that insufficient competencies evoked feelings of insecurity and inadequacy in novice nurses, affecting their transition [43]. However, studies suggest that combining didactic content, clinical content and preceptors can increase novice nurses’ confidence and promote successful transition [25,32,62].

Preceptors can provide professional advice and develop and validate the competencies of novice nurses, leading to increased confidence and an enhanced transition process [43]. Novice nurses voiced a need to pair with experienced preceptors for guidance and support via positive feedback and remedial advice. Preceptors' positive, constructive feedback and remedial advice help develop novice nurses’ knowledge and skills and are critical for their success [10]. Through professional advice and validation of competencies, preceptors can enhance the transition of novice nurses in unique ED environments, thereby contributing to a supportive learning environment. Preceptors can subsequently contribute to a safe, positive learning environment, which is pivotal to ensuring the transition into EDs [16,25].

Our participants reported having experienced unpleasant feelings and teamwork in the ED and wished for a positive learning environment. A hostile learning environment is a barrier to transition and is characterised by poor teamwork, hostility, and unsupportive ED staff [36]. The ED environment exposes novice nurses to heavy workloads, uncertainty concerning patient care, and repeated trauma situations, which contribute to occupational stress [15]. Emotional support may reduce occupational stress and improve job satisfaction [15]. The novice nurses in our study highlighted the importance of addressing emotional support through one-to-one support sessions, support groups, regular debriefing sessions and counselling by psychologists [50]. The incorporation of novice nurses’ support needs may inform the development of evidence-based programs to support transition in specific contexts [18]. Although most studies have focused on educational needs and the availability of preceptors for a successful transition [4,28,33], our study highlighted the importance of a healthy, safe and supportive learning and working environment for a successful transition.

## Limitations

This study has several limitations. The limitations include restricted generalizability due to the small, purposively selected sample from the urban hospitals in a district of South Africa. Furthermore, the reliance on thematic analysis means that findings are deeply tied to the authors' interpretative skills, which could lead to variability in identified themes.

## Conclusion

The successful transition of novice nurses in EDs is a cost-effective way of improving job satisfaction, retention, and recruitment. Novice nurses working in EDs have diverse support needs for smooth transitions, and this data suggests the need for a dedicated orientation programme, dedicated preceptors, and a supportive culture to facilitate the transition to the ED. A dedicated orientation programme should include emergency equipment, triage, BLS skills, admission and treatment protocols and specific conditions. Access to dedicated preceptors who are ED trained and experienced and who can provide feedback and remedial support to novice nurses is valuable. Moreover, a supportive ED culture characterised by a positive learning environment and emotional support can facilitate the transition of novice nurses in this environment. Addressing novice nurses’ support needs can positively affect nurses’ competence and confidence, patient care outcomes, job satisfaction and retention. Future research should focus on the support needs of novice nurses in more diverse contexts and on experienced and trained nurses’ inputs regarding transitional support in EDs.

## Dissemination of results

The results from this study have been shared with the hospitals involved and at various emergency nursing conferences and social media platforms.

## CRediT authorship contribution statement

**Kapari Mashao:** Conceptualization, Methodology, Data curation, Formal analysis, Investigation, Writing – original draft. **Celia Filmalter:** Supervision, Validation, Writing – review & editing. **Tanya Heyns:** Conceptualization, Methodology, Data curation, Formal analysis, Supervision, Validation, Writing – review & editing.

## Declaration of competing interest

The authors declare that they have no known competing financial interests or personal relationships that could have appeared to influence the work reported in this paper.

## Appendix Supplementary materials

Image, application 1

## Footnotes

Supplementary material associated with this article can be found, in the online version, at doi:10.1016/j.afjem.2025.100889.
