# Supplementary material for: Perceived support needs of novice nurses working in emergency departments of selected public hospitals in Tshwane, South Africa
Source: Afr J Emerg Med. 2025 Jul 23;15(3):100889. doi: 10.1016/j.afjem.2025.100889 (PMC12305558; doi:10.1016/j.afjem.2025.100889)
Supplement: Supplementary file 1 [file mmc1.docx]

**Supplemental material: Summary of competencies required**

| **Competencies** | **Supportive quotes** | **Supportive resources** |
| --- | --- | --- |
| Assessment of patients | *“…as* [novice] *nurses working in emergency department, we need to perform proper assessment of patients…”* (P14) | Ndung’u et al. (2022) [14] |
| Electrocardiogram interpretation | *“…I think if we can get teaching about ECGs, if we can know the different types of complication of ECGs. We might see certain conditions and be able to diagnose them before we can refer to a doctor and know how to approach certain ECGs…We need to know about X-rays. How to differentiate if this is hemothorax? Or having pneumothorax... …”* (P15) | Rahimpour et al. (2021) [25]) |
| Mechanical ventilation | *“…we never learned how to manage a patient on a* [mechanical] *ventilator…”* (P11) | Rafiq et al. (2021) [26] |
| Arterial blood gas analysis | *“…Sometimes blood gas is just a bunch of numbers and people don’t understand the implication of how to interpret…”* (P5) | Vahedian-Azimi et al. (2021) [27] |
| Inserting intravenous and urine catheter | *“There are people* [novice nurses] *that are actually still struggling with inserting of a drip* [intravenous catheter] *and urine catheter… The stitches, suturing the stitches that is also a challenge…”* (P9) | Burchill et al. (2021) [28]  Bhatt et al. (2021) [29] |
| Management of patient admitted with trauma | *“…How to* [novice nurses] *manage trauma, a newly fresh, P1 trauma case. How to approach, what to do when this kind of trauma case comes in… and we can do training regarding polytraumas…”* (P15) | Ndung’u et al. (2022) [14] |
| Medical emergencies | *“…Like how to manage DKA* [diabetes ketoacidosis]*, hypoglycemia, hypertension in the emergency department…even also to work with mental healthcare users… and the pediatrics* [pediatric patients]*. Some of the patients are pregnant, so we must be taught how to assess a pregnant woman...”* (P14) | Dulandas & Brysiewicz (2018) [4] |
| Mental healthcare users | *“…Our ED department works a lot with psychiatric [mental healthcare user], especially violent psychiatric [mental healthcare user] patients. So just methods of restraining, sedating and how to work with violent psychiatry patient…” (P7)* | Chou & Tseng (2020) [30] |
| Interpret chest X-rays | *We need to know about X-rays. How to differentiate if this is hemothorax…or having pneumothorax...…”* (P15) | Dulandas & Brysiewicz (2018) [4] |
| Basic life support: Adults and pediatrics | *“…So, I think it's important as well to go into the resus of babies knowing that you can't take the same doses of medication to adults and babies. And that babies need other support than adults…”* (P7) | Phukubye et al. (2021) [31] Dulandas & Brysiewicz (2018) [4] |
| Suturing | *…the stitches…suturing the stitches that is also a challenge…”* (P9) | Şimşek et al. (2020) [32] |
| Assist with advanced procedures | *“… assist the doctor with intubation… this is the knowledge that is required. Those are the things that I start knowing while I'm here at emergency department…”* (P3) | Ndung’u et al. (2022) [14] |
| Medication indications and administration | *“…And knowledge of medication cause now in a critical situation you will be using medication which you are not using in normal situations. I think if new nurses are taught that it could make a difference…”* (P8)  *“…You have to give medications, you have to know the inotropes you have to know the sedation, you have to know how the antibiotics work…”* (P3)  *“…And the drugs that are in the drug cupboard. It's a lot of drugs. Yeah. And some of them have similar names, so it's very easy to make a mistake…”* (P10)  *“…Oh and calculating medication for the babies… I think also that is also a challenge most of the times…”* (P9) | Shitu et al. (2020) [33] |
| Documentation | *“…And then another important thing is documentation. That is the biggest one. How do I use documents in a way that I make sure that I covered all my bases with the patient included? “…How do we document in the emergency department? How do we write our cardex? Can we be efficient without having to write a two-page cardex? How can I include the necessary information that is comprehensive…So, I think for the first two days, maybe two to three days we look at things like documentation…”* (P1) | Gupta et al. (2020) [34] |
| Participate in interprofessional team | *“Teamwork is very key, like is very important in casualty. You can’t resus alone…”* (P5)  *“And when I got here, I didn’t get what we call teamwork. It was like everyone [emergency department nurses] where in their corners. Then, even though I went through that thing [lack of teamwork] I had to take it positive and it’s part of building the person I am today. But I think teamwork is very important because when at least you are with someone [registered nurse] who’s used to the area and how things are done is able to give you a colleague moral support.” (P6).* | Olde-Bekkink et al. (2018) [35] |
